# Family systems approach to attachment relations, war trauma, and mental health among Palestinian children and parents

**DOI:** 10.1080/20008198.2018.1439649

**Published:** 2018-03-20

**Authors:** Raija-Leena Punamäki, Samir R. Qouta, Kirsi Peltonen

**Affiliations:** a Faculty of Social Sciences Psychology, University of Tampere, Tampere, Finland; b Department of Education and Psychology, Islamic University Gaza, Gaza City, Palestine

**Keywords:** War trauma, attachment styles, post-traumatic stress disorder (PTSD), depression, families, Palestinian, trauma de guerra, estilos de apego, trastorno por estrés postraumático (TEPT), depresión, familias, palestinos, 战争创伤, 依恋风格, 创伤后应激障碍（PTSD）, 抑郁, 家庭, 巴勒斯坦, • Diversity of attachment and sibling relationships characterizes families in war conditions, showing both resilience and vulnerabilities.• Children in families with secure attachment, warm sibling relationships, and optimal parenting show good mental health and functional trauma processing.• Psychosocial interventions among war-affected children should also improve family relationships, e.g. by increasing secure attachment and by decreasing conflicts and rivalry in siblingship.

## Abstract

**Background**: Trauma affects the family unit as a whole; however, most existing research uses individual or, at most, dyadic approaches to analyse families with histories of trauma.

**Objective**: This study aims to identify potentially distinct family types according to attachment, parenting, and sibling relations, to analyse how these family types differ with respect to war trauma, and to explore how children’s mental health and cognitive processing differ across these family types.

**Method:** Participants included Palestinian mothers and fathers (*N* = 325) and their children (one per family; 49.4% girls; 10–13 years old; mean ± *SD* age = 11.35 ± 0.57 years) after the Gaza War of 2008–2009. Both parents reported their exposure to war trauma, secure attachment availability, and parenting practices, as well as the target child’s internalizing and externalizing symptoms [Strengths and Difficulties Questionnaire (SDQ)]. Children reported their symptoms of post-traumatic stress disorder (on the Children’s Revised Impact Event Scale), depression (Birleson), and SDQ, as well as their post-traumatic cognitions (Children’s Post Traumatic Cognitions Inventory).

**Results:** A cluster analysis identified four family types. The largest type reflected secure attachment and optimal relationships (security and positive family relationships, 36.2%, *n* = 102), and the smallest exhibited insecurity and problematic relationships (insecurity and negative family relationships, 15.6%; *n* = 44). Further, families with discrepant experiences (23.0%; *n* = 65) and moderate security and neutral relationships (25.2%; *n* = 71) emerged. The insecurity and negative relationships family type showed higher levels of war trauma; internalizing, externalizing, and depressive symptoms among children; and dysfunctional post-traumatic cognitions than other family types.

**Conclusion:** The family systems approach to mental health is warranted in war conditions, and therapeutic interventions for children should, thus, also involve parents and siblings. Knowledge of unique family attachment patterns is fruitful for tailoring therapeutic treatments and preventive interventions for war-affected children and families.

## Abstract (es)

**Objetivo**: el trauma afecta a la unidad familiar como un todo; sin embargo, la mayoría de las investigaciones existentes utilizan abordajes individuales o, a lo sumo, diádicos para analizar familias con historias de trauma. El objetivo de este estudio es identificar tipos de familia potencialmente distintos en función de las relaciones de apego, de crianza y entre hermanos; analizar cómo difieren estos tipos de familia con respecto al trauma de guerra; y explorar cómo la salud mental y el procesamiento cognitivo de los niños difieren entre estos tipos de familia.

**Métodos**: Los participantes incluyeron madres y padres palestinos (*N* = 325) y sus hijos (uno por familia, 49.4% niñas, 10-13 años, M = 11.35 ± 0.57) después de la Guerra de Gaza de 2008 a 2009. Ambos padres comentaros su exposición a los traumas de guerra, su disponibilidad para ofrecer un apego seguro y sus prácticas de crianza, así como a los síntomas de internalización y externalización del niño (*Strengths and Difficulties Questionnaire*, SDQ; Cuestionario de fortalezas y dificultades). Los niños indicaron sus síntomas de trastorno de estrés postraumático (TEPT, CRIES), depresión (Birleson) y SDQ, así como sus cogniciones postraumáticas (CPTCI).

**Resultados**: Un análisis de*clusters* identificó cuatro tipos de familia. El tipo más numeroso reflejaba apego seguro y relaciones óptimas (seguridad y relaciones familiares positivas, 36.2%, *n* = 102), y el tipo menos numeroso mostraba inseguridad y relaciones problemáticas (inseguridad y relaciones familiares negativas, 15.6%, *n* = 44). Además, aparecieron familias con experiencias discrepantes (23.0%, *n* = 65), con seguridad moderadas y relaciones neutrales (25.2%, *n* = 71). El tipo de familia con inseguridad y relaciones negativas mostró niveles más altos de trauma de guerra; síntomas de internalización, externalización y depresión entre los niños; y cogniciones postraumáticas disfuncionales que otros tipos de familia.

**Conclusión**: El abordaje de sistemas familiares para la salud mental está justificado en condiciones de guerra, y las intervenciones terapéuticas para los niños deberían, por tanto, implicar también a padres y hermanos. Conocer los patrones de apego familiar particulares es fructífero para adaptar los tratamientos terapéuticos y las intervenciones preventivas para niños y familias afectados por la guerra.

## Abstract (zh)

**目标**：创伤的影响是以家庭为单位的；但大多数现有的研究仅使用个体咨询或者最多双方咨询来分析有创伤历史的家庭。本研究旨在根据依恋、教养和同胞关系识别潜在不同的家庭类型，然后分析这些家庭类型如何随战争创伤不同，以及探索儿童的精神健康和认知加工如何在这些家庭类型间不同。

**方法**：参加者包括经历加沙战争后2008到2009年的巴勒斯坦父母(*N* = 325)和子女（每个家庭一人，49.4% 女孩; 10–13 岁; M = 11.35 ± 0.57）。两位父母都报告他们的战争创伤经历，安全依恋风格和教养方式，也包括孩子的内化和外化问题（《力量和问题问卷》，SDQ）。儿童报告他们的创伤后应激障碍（PTSD, CRIES）症状，抑郁（Birleson问卷），SDQ和他们的创伤后认知（CPTCI）。

**结果**：聚类分析识别四个家庭类型。最大的类型组反映了安全依恋和最好的关系（安全和积极关系——36.2%的家庭，*n* = 102）。最小的组展示了不安全和问题关系（不安全和负面关系——15.6%家庭，*n* = 44）。还有，不一致体验的家庭（23.0%; *n* = 65）和中等安全/关系(25.2%; *n* = 71) 。不安全和负面关系家庭类型和其它类型相比，显示了更高水平的战争创伤；儿童的内化、外化和抑郁症状；非功能性的PTC。

**结论**：家庭系统方法应对精神健康在战争情景中是可以担保的，所以针对儿童的治疗性干预应该让家长或者兄弟姐妹加入。对独特的家庭依恋模式的了解成效显著地让咨询式治疗和战争影响的儿童和家人的预防性干预变得个性化。

## 1. Introduction

When discussing families living in conflict zones, parents commonly express worry about their children’s safety, and children frequently refer to their family members as a source of security. A Palestinian father said, ‘My 3-year-old promised to guarantee electricity to our whole family when she grows up. I felt ashamed of my inability.’ ‘Without the help of my bigger sister, I would be dead,’ a Palestinian boy said after the Gaza War of 2008–2009. Research has confirmed that families face hardships together, forming a system in which each member takes on an ‘emotional share of work,’ showing endurance, manifesting symptoms, and caring for one another (Barajas-Gonzalez & Brooks-Gunn, 2014; Crittenden & Dallos, 2009; Montgomery, 2004). Research on the transgenerational transition of trauma has indicated both vulnerability, often reflected in relational problems, social withdrawal (Daud, Skoglund, & Rydelius, 2005), and psychiatric symptoms, such as post-traumatic stress disorder (PTSD) (Yehuda & Bierer, 2008), and resilience, which manifests as caring and empathy towards the traumatized family members (Fossion et al., 2015).

According to the family systems approach, families are composed of multiple dynamically interacting relational subsystems typically involving marital, parent–children, and sibling relationships (Coyne, Downey, & Boergers, 1992; Minuchin, 1974). Although traumatic experiences, such as war, influence all family members, few studies have focused on the whole family as the unit of research. Instead, studies on trauma-affected families have typically applied a variable-oriented approach, analysing separate characteristics, such as parenting quality or attachment styles, in associating with family members’ mental health (Dekel & Monson, 2010). A person-oriented approach, by contrast, can identify unique homogeneous family patterns and depict the dynamic and multiple relationships among family members in different subsystems (Bergman & Magnusson, 1997). In the current study, we apply a person-oriented approach (cluster analysis) to identify family types with different attachment, parenting, and sibling dynamics among Palestinians who live in the midst of political and military conflict in Gaza. We also analyse the role of war trauma as a predictor of family type and explore how children’s mental health and cognitive processing differ across these types.

### 1.1. Family systems and attachment research

System theories conceptualize families according to their structure (e.g. boundaries between subsystems, dominance hierarchies, and communication transparency) and relational context (e.g. autonomy vs intimacy; harshness vs warmth) (Blass & Blatt, 1992; Kerig, 2005; Olson, 2000). Research has delineated, for instance, enmeshed families, characterized by too thin boundaries with easily evoked emotional spillover between parents and children, and disengaged families, characterized by too thick boundaries and a lack of support and emotional closeness among members. Cohesive families, in turn, enjoy a balance between autonomy and intimacy, meaning that members have access to both emotional support and individuality and privacy (Kerig, 2005; Lindblom et al., 2017; Minuchin, 1974).

Attachment theory is highly informative for understanding family relations in cases of war and other traumatic conditions. A sense of security is a core motivator for young children, and the goodness of fit among the emotional, cognitive, and behavioural responses of parents and children is essential to survival and mental health (Bowlby, 1969/1982). In secure families, children learn to trust themselves and to seek shelter with their caregivers. They dare to express both positive and negative emotions and develop working models in which they see themselves as capable, others as reliable, and their environment as predictable (Bowlby, 1969/1982). A secure attachment relationship with a sensitive and emotionally available caregiver provides a child with a safe base and supports a balance between exploring and emotional holding. By contrast, children with an insecure–avoidant style seek protection in themselves because they fail to receive security from emotionally distant caregivers. Insecure–ambivalent children try to create a feeling of safety by clinging to caregivers and other adults, who are often emotionally ambivalent and unpredictable. Ample evidence confirms the importance of a sense of security in optimal child development and mental health (Pallini, Baiocco, Schneider, Madigan, & Atkinson, 2014).

### 1.2. Trauma and family dynamics

Models integrating attachment and family systems theories conceive of triadic, dyadic, and individual experiences as developing within the larger attachment and family–cultural networks (Crittenden & Dallos, 2009; Masten & Monn, 2015). Research has found complex dynamics in attachment relations and identified several ways that family members show vulnerability and resilience in the face of traumatic events (Besser & Neria, 2010; Freedman, Gilad, Ankri, Roziner, & Shalev, 2015). A family’s atmosphere, values, beliefs, relational scripts, codes, histories, and emotional sharing all influence family members’ responses to trauma (Riggs & Riggs, 2011; Walsh, 2007). Family systems theories provide insight into family members’ dynamic social and mental health responses to stress, including compensatory, buffering, and additive dynamics (Davies & Cicchetti, 2004; Minuchin, 1974). A study of war-affected Palestinian families revealed that parents and siblings take on a compensatory emotional ‘share of the work’ when expressing mental health problems and resilience (Punamäki, Qouta, El Sarraj, & Montgomery, 2006). For instance, when mothers showed high levels of depressive symptoms, fathers reported low levels. Similarly, when one sibling was a ‘symptoms carrier’ (e.g. when one sibling suffered from severe PTSD symptoms), others exhibited better adjustment. This sharing of the emotional work was especially evident in families exposed to severe war trauma.

Family systems and attachment-informed research is available on military families, especially American veterans of the Iraq and Afghanistan wars (Pemberton, Kramer, Borrego, & Owen, 2013; Riggs & Riggs, 2011) and Israeli soldiers, veterans, and prisoners of war (Cohen, Zerach, & Solomon, 2011; Ein-Dor, Doron, Solomon, Mikulincer, & Shaver, 2010; Zerach, Greene, Ein-Dor, & Solomon, 2012). These studies found negative family dynamics, especially when the veteran parents had PTSD. The families with a traumatized parent typically suffered a low sense of security, biased or narrow family attachment networks, relational rigidity with cemented roles of strength and weakness, and scapegoating.

Transgenerational research on Holocaust (Van Ijzendoorn, Bakermans-Kranenburg, & Sagi-Schwartz, 2003) and torture (Montgomery, 2011) survivors provides further information on the dynamics of families affected by trauma. Parental past trauma has been found to be associated with PTSD, depression, and somatic complaints among children (Montgomery & Foldspang, 2005; Yehuda & Bierer, 2008). Poor parenting and siblingship, as well as biased and silenced family communication, can underlie children’s vulnerability to parental trauma (Bryant, 2016; Frewen, Brown, DePierro, D’Andrea, & Schore, 2015; Schierholz, Kruger, Barenbrugge, & Ehring, 2016).

Traumatized parents can be either overprotective (owing to fears and concerns for their children’s safety) or unable to tolerate their children’s manifestations of fear, helplessness, and neediness (Scheeringa & Zeanah, 2001). Thus, parents may adopt intrusive and insensitive child-rearing practices, typical of persons with preoccupied attachment styles, or may easily withdraw from dyadic interactions, typical of persons with avoidant attachment styles (Flykt, Kanninen, Sinkkonen, & Punamäki, 2010; Van Ee, Kleber, Jongmans, Mooren, & Out, 2016). A qualitative study confirmed that parental refugee trauma disturbed children’s creation of secure attachments because the parental fears were overwhelming (De Haene Grieten, & Verschueren, 2010). In a follow-up setting of Israeli families with traumatic war experiences, Besser and Neria (2010) showed that parental insecure attachment predicted severe depressive symptoms and poor social support, both of which can compromise optimal parent–child relations (Frewen et al., 2015).

By contrast, supportive, secure, and wise parenting practices can protect children’s mental health, optimal development, and resilience in the life-endangering conditions of war (Betancourt et al., 2011; Cummings et al., 2011; Feldman, Vengrober, Eidelman-Rothman, & Zagoory-Sharon, 2013; Qouta, Punamäki, Miller, & El Sarraj, 2008). A study of Northern Irish children and parents confirmed that secure emotional family relations predicted low levels of psychological distress despite prolonged and severe sectarian and political violence (Cummings et al., 2011). Follow-up analyses revealed close and dynamic interactions among traumatic political events, children’s aggressive symptoms, and family violence and conflicts. Children’s community-evoked emotional insecurity made them more vulnerable when facing family conflicts, and vice versa (Cummings, Taylor, Merrilees, Goeke-Morey, & Shirlow, 2016).

### 1.3. Trauma, child development, and well-being

There is ample evidence of war trauma negatively affecting children’s mental health by increasing symptoms of PTSD, depression, anxiety, and aggression (Attanayake et al., 2009; Dubow et al., 2009). Less is known about war trauma’s impacts on children’s attachment styles, although, theoretically, trauma is assumed to increase the risk of insecure attachment. Childhood traumas of neglect, deprivation, and socioeconomic hardship predict the development of insecure attachments (Scheeringa & Zeanah, 2001), and some research shows a higher level of insecure attachments among traumatized children and adolescents (Huemer et al., 2016; Turunen, Haravuori, Punamaki, Suomalainen, & Marttunen, 2014).

It is generally agreed that it is not exposure to war trauma alone, but also survivors’ cognitive–emotional processing of their experiences that contribute to mental health consequences (Ehlers & Clark, 2000; Schnyder et al., 2015). According to cognitive theories, thinking, attributions, appraisals, and beliefs about traumatic events are decisive for the emergence and maintenance of mental health problems like PTSD or depression (Ehlers & Clark, 2000; Meiser-Stedman, 2002). A trauma victim’s negative post-traumatic cognitions (PTCs) depict the core of his or her perceptions of himself or herself as helpless and worthless, of other people as malevolent and distrustful, and of the community as dangerous and unpredictable (Foa, Ehlers, Clark, Tolin, & Orsillo, 1999; Meiser‐Stedman et al., 2009). Research has confirmed that overly negative appraisals, dysfunctional memories, supressed emotions, and feelings of guilt and anger predict PTSD in children (Trickey, Siddaway, Meiser-Stedman, Serpell, & Field, 2012). Many child- and trauma-related factors predict dysfunctional and negative PTCs (Ehlers, Mayou, & Bryant, 2003). However, we found no studies on how attachment or other family relationships contribute to the ways in which children process their traumatic experiences, which is the topic of the current research.

### 1.4. Research questions

First, our study aims to identify distinct family types according to mother, father, and child attachment responses; sibling subsystems; and parenting among families living in war conditions. Secondly, we examine how the family types differ in their exposure to traumatic war events, as indicated by human losses, material destruction, the witnessing of horrors, and the experiencing of life-threatening situations during the Gaza War. Thirdly, we analyse how family type is associated with children’s mental health, as assessed by PTSD, depressive, and internalizing and externalizing symptoms. Fourthly, we analyse whether children’s PTCs differ across the identified family types.

## 2. Method

### 2.1. Participants and procedure

Palestinian families (*N* = 325), each with a mother, a father, and one 10–13-year-old child (mean age = 11.35 years, *SD* = 0.57 years; 49% girls), participated in May 2009, 3 months after the Gaza War. This is a baseline subsample of a larger randomized intervention study (*N* = 482) that examined the effectiveness of a post-war psychosocial intervention programme (Qouta, Palosaari, Diab, & Punamäki, 2012). In the family subsample, both mothers (*n* = 337) and fathers (*n* = 328) responded, and their children participated in either the intervention group or the control group. The analysed data are from the 325 participating families for which both parents provided information to support the analysis of their family dynamics.

The return rates for the mothers’ and fathers’ questionnaires were 69.9% and 68.0%, respectively. The family subsample of 325 did not differ from the families who did not participate (*n* = 157) in terms of fathers’ or mothers’ work status [respectively, *χ* ^2^(1) = 0.38, *p* = ns and *χ* ^2^(1) = 0.01, *p* = ns], place of residence [*χ* ^2^(1) = 0.28, *p* = ns], family structure [*χ* ^2^(2) = 2.3, *p* = ns], or size [*χ* ^2^(2) = 0.11, *p* = ns]. However, the participating families were biased towards having more girls as the target child (56.1%) than the families that did not participate (35.2%) [(*χ* ^2^(1) = 17.72, *p *< 0.0001].

The original baseline sample represented the regions of the Gaza Strip that were severely bombed during the Gaza War. First, eight schools were randomly selected from 160 potential schools in these regions, taking into consideration that girls and boys go to separate schools. Then, from each of these eight schools, one sixth-grade and one seventh-grade class were randomly chosen, resulting in 16 classes whose pupils participated in the baseline assessment that serves as the data for the current cross-sectional analysis.

The ethics boards of the Palestinian Ministry of Education and the Gaza Community Mental Health Program (GCMHP) reviewed and accepted the study’s protocols and measurements, and permission for the study was received from the schools’ authorities. Information sheets were provided to children and their parents explaining the procedure of the study, but parents only gave verbal consent for their children to participate in the study and in the psychosocial intervention. Six research assistants collected the children’s data in the classroom. The children took the parents’ questionnaires home to complete and returned them in closed envelopes to the research assistants. The second author (SQ) supervised the data collection through weekly sessions and school visits.

### 2.2. Measures

#### 2.2.1. Parental attachment security

This was measured using the 10-item Security Scale (Kerns, Klepac, & Cole, 1996), which depicts a parent’s acceptance of and willingness to serve as an attachment figure and provide a secure base for a child. The items include, for example, ‘I respect my child’s opinions and encourage him/her to express them,’ ‘I feel a child should be given comfort and understanding when she/he is scared or upset,’ and ‘I make sure my child knows that I appreciate what she/he tries to accomplish.’ Mothers and fathers responded by noting how well each item corresponded to their attitudes and behaviours towards the target child on a six-point Likert scale (1 = not at all; 6 = very well). The resulting sum variables showed moderate reliability. The Cronbach’s *α* values were 0.69 for mothers and 0.68 for fathers.

#### 2.2.2. Parents’ war trauma

The 28-event checklist for Gaza War-related traumatic experiences covered personal exposure to war trauma (e.g. detained, tortured), material destruction (e.g. shelled/bombed neighbourhood, home demolished by the military), family losses (e.g. family member killed, family separation), and witnessing horrors (e.g. witnessing a killing, seeing body parts). Mothers and fathers reported whether they had experienced these events during the war (1 = yes; 0 = no). Four sum variables were constructed for both parents by counting the positive answers relating to personal exposure to trauma (seven events), material destruction (nine events), family losses (five events), and witnessing horrors (seven events).

#### 2.2.3. Negative parenting

The 20-item Child Psychological Maltreatment questionnaire (Khamis, 2000) covers emotional abuse (seven items, e.g. ‘My parents humiliate me in front of people’), emotional neglect (seven items, e.g. ‘My parents ignore my attempts to interact with them’), and harsh parenting (six items, e.g. ‘My parents force me to do things and tasks against my will’). Children and parents evaluated how well the descriptions fitted their parents (children) or their own behaviours and rearing practices on a five-point Likert scale (1 = strongly disagree; 5 = strongly agree). Three averaged sum variables were constructed for the children (*α* = 0.94 for emotional abuse, *α* = 0.89 for emotional neglect, and *α* = 0.53 for harsh parenting) and parental variables by combining the responses of the mothers and fathers (*α* = 0.94 for emotional abuse, *α* = 0.89 for emotional neglect, and *α* = 0.73 for harsh parenting).

#### 2.2.4. Children’s attachment style

Children’s attachment style was measured using the Coping Strategies Questionnaire (CSQ) (Finnegan, Hodges, & Perry, 1996) and the Security Scale (Kerns, Tomich, Aspelmeier, & Contreras, 2000). Together, these distinguish 28 everyday situations to measure avoidant, preoccupied, and secure attachment. The children’s responses reflected their mothers as attachment figures who helped them, listened to them, and cared for them daily during stressful situations. Their answers were depicted using two-stage methods. For example, ‘avoidant attachment’ was assessed by 10 everyday situations, such as, ‘One day you come home from school, and you are upset about something. Your mother asks you what the problem is.’ For each situation, respondents chose between two-stage forced choices: (1) talk to her about it or (2) not talk her about it. Underneath these two choices were two more alternatives: (a) sort of true for me or (b) very true for me. ‘Preoccupied attachment’ was also assessed by 10 everyday situations, such as ‘Your mother says she is thinking about going to visit a relative for a week.’ Here, the two choices were (1) being upset that she was going away for so long and trying to talk her out of going and (2) not being upset and not trying to talk her out of going. Again, two further sub-choices were presented: (a) very true for me and (b) sort of true for me (reverse coding). In measuring ‘felt security attachment’, the children were instructed to answer the question ‘How do you feel about your mother?’ by selecting from eight provided two-stage choices, such as: ‘Some kids worry that their mom might not be there when they need her’ (but) ‘Other kids are sure their mom will be there when they need her,’ and ‘Some kids feel that their mom does not help them enough with their problems’ (but) ‘Other kids think that their mom helps them enough with their problems.’ Averaged sum variables were formed for felt security (*α* = 0.66), avoidant (*α* = 0.63), and preoccupied (*α* = 0.54) attachments, and showed low reliabilities.

#### 2.2.5. Sibling relations

The quality of siblingship was assessed using an 11-item scale describing positive (warmth and intimacy) and negative (conflict and rivalry) interactions (Dunn, Slomkowski, & Beardsall, 1994). Children estimated how often the described events happen in their relations with older (11 items) and younger (11 items) siblings using a five-point scale (1 = never; 5 = always). All items for older and younger sibling relationships correlated significantly, and averaged composite variables were calculated by combining the items for both siblings: warmth in siblingship (e.g. ‘We laugh and joke together’ or ‘I miss him/her when he/she is out of the home’), intimacy (‘I tell him/her about my secrets’ or ‘I play and share games with him/her’), conflicts (‘He/she annoys and teases me’ or ‘At times, he/she beats me and pushes me’) and rivalry (‘I feel jealous of him/her when he/she takes all my mother’s attention’ or ‘I feel unhappy or jealous when other children play with him/her and ignore me’). Averaged sum variables were constructed with reasonable reliabilities (Cronbach’s *α* values were 0.72, 0.68, 0.75, and 0.79, respectively).

#### 2.2.6. Children’s post-traumatic stress symptoms (PTSD)

PTSD symptoms were evaluated using the 13-item Children’s Revised Impact Event Scale (CRIES) (Dyregrov, Gjestad, & Raundalen, 2002). The scale covers the three core symptoms of re-experiencing (four items), avoidance (four items), and hyperarousal (five items). Children indicated on a four-point Likert scale (0 = not at all; 4 = often) how often they had experienced each symptom during the last 2 weeks. A total score was constructed, and the Cronbach’s *α* value was low at 0.61.

#### 2.2.7. Children’s depression

The Depression Self-Rating Scale for Children (Birleson, Hudson, Grey-Buchanan, & Wolff, 1987) is an 18-item self-report assessment of the cognitive, affective, and behavioural dimensions of depression. Children estimated on a three-point scale (0 = not at all; 1 = sometimes; 2 = all the time) whether they had experienced each symptom during the last 2 weeks. A sum score of the depression symptoms was then formed with a Cronbach’s *α* value of 0.78.

#### 2.2.8. Children’s externalizing and internalizing symptoms

The Strengths and Difficulties Questionnaire (SDQ) (Goodman, 1997) uses five behavioural descriptions each to assess hyperactivity, prosocial behaviour, and emotional, behavioural, and relational problems. Both parents and children estimated on a three-point Likert scale (0 = not at all; 1 = somewhat; 2 = yes, fits well) how well the description fitted the target child (or the child her/himself). Sum scores were constructed for the parents’ reports by combining the mothers’ and fathers’ scores (Cronbach’s *α* values for parents’ internalizing and externalizing symptoms were 0.73 and 0.79, respectively) and for the children’s reports (Cronbach’s *α* values for children’s internalizing and externalizing symptoms were 0.71 and 0.69, respectively).

#### 2.2.9. Children’s post-traumatic cognitive appraisals

These appraisals were measured using the 25-item Children’s Post Traumatic Cognitions Inventory (CPTCI) (Meiser‐Stedman et al., 2009). The items include statements relating to negative appraisals of the world and the self. The dimensions are (1) the trauma-exposed child as a feeble person in a scary world (e.g. ‘Anybody could hurt me’ or ‘I can’t stop bad things from happening to me’) and (2) the life after trauma, involving disturbing and permanent negative change (e.g. ‘My life has been destroyed by the frightening event’ or ‘Not being able to get over all my fears means that I am a failure’). Children evaluated on a four-point Likert scale their agreement with each statement (1 = don’t agree at all; 2 = don’t agree a bit; 3 = agree a bit; 4 = agree a lot). A sum variable was constructed for CPTCI scores with an *α* value of 0.85.

#### 2.2.10. Children’s war trauma

A checklist of 28 war-related events was constructed of typical experiences during the Gaza War and military occupation (UN, 2009). The checklist covered child-targeted violence (e.g. being wounded, beaten, or burned by phosphorous bombs), family-related losses (e.g. death of father, mother, siblings, or friends; loss of home; and being separated during the war), witnessing horrors (e.g. witnessing people dying and being injured and seeing body parts), and material destruction (e.g. neighbourhood shelled/bombed, home demolished, or besieged). Children reported whether they had been exposed to each event (1 = yes; 0 = no) either during the war or earlier. War trauma is a linear sum variable reflecting the total number of all ‘yes’ answers.

#### 2.2.11. Demographic variables

Mothers and fathers reported family income, parental education, work situation, family size, family structure (extended or core), and children reported their  age and gender.

#### 2.2.12. Translations

The research instruments for the CRIES-13, Birleson depression, and war trauma scales were available in Arabic. The children’s and parents’ attachment scales and PTCs (CPTCI) were first translated by a bilingual psychologist from English into Arabic and then translated back by the research group.

### 2.3. Statistical analyses

Cluster analyses were used to identify distinct family type subgroups based on the variables of siblingship, parenting, and mother, father, and child attachment. We first ran a hierarchical cluster analysis to define the number of groups and then ran a *K*-means cluster analysis to confirm the cluster membership. The dendrogram inspection in the hierarchical clustering helped us to choose the number of initial clusters. *K*-means clustering produces a cluster centre (centroid) initialization and a squared Euclidean distance measure, which are used to search for the location of each case within the defined family clusters. Before the cluster analyses were run, the variables were standardized into *t*-scores to avoid biases due to differences in scales (Tabachnik & Fidell, 2007). Cluster membership was tested by multivariate analyses of variance (MANOVAs) using univariate analyses and Tukey-b post-hoc tests.

To analyse how the identified family types were associated with families’ exposure to war trauma, we ran the MANOVA with the identified cluster family type as an independent variable and father- and mother-reported war trauma (e.g. personal exposure to trauma, material destruction, family losses, and the witnessing of horrors) as the dependent variables. Further, to analyse the associations between family types and children’s mental health, we ran a multivariate analysis of covariance (MANCOVA) with parent- and child-reported internalizing and externalizing symptoms and child-reported depressive and PTSD symptoms as independent variables. A MANCOVA was also used to analyse the family types associated with post-traumatic cognitions (CPTCI), with being a feeble person in a scary world and permanent negative change as the independent variables. Children’s gender and children’s war trauma were the covariates. The MANCOVAs were followed by univariate analyses and Tukey-b post-hoc tests.

## 3. Results

### 3.1. Descriptive statistics

Table 1 shows the demographic characteristics reported by the parents and their children. About a quarter (24%) of the fathers had a university education, while less than 10% of the mothers had one. Despite their education, about half (49%) of the fathers were unemployed, and nearly all (93%) of the mothers worked at home. These statistics correspond with the economic and social situation in Gaza, which deteriorated following the Israeli military siege and international boycott of the Hamas government (UN:OCHA, 2009). Nearly a third (29%) of the participants lived in extended families, and family size was large: about a quarter (26%) had more than eight children.

**Table 1.** Percentages and frequencies of demographic family factors.

	%	*n*
Place of living		
City	84.3	284
Refugee camp	3.3	11
Village	12.5	42
Status		
Refugee	11.3	38
Citizen	88.7	299
Mother’s education		
Elementary	19.6	66
Preparatory	32.4	109
Secondary	39.9	134
University	8.0	27
Father’s education		
Elementary	21.1	71
Preparatory	28.3	95
Secondary	26.2	88
University	24.4	82
Father’s work situation		
Unemployed	49.3	166
Worker	12.8	43
Public employee	24.9	84
Entrepreneur/self-employed	13.1	44
Mother’s work situation		
Works at home	93.2	314
Worker	3.0	10
Public employee	3.9	13
Other		
Family type		
Immediate	61.9	210
Extended	28.9	98
Tribe	9.1	31
Family size		
Small (1–4)	23.8	80
Medium (5–7)	50.0	168
Large (8 or more)	26.2	88

**Figure 1.**
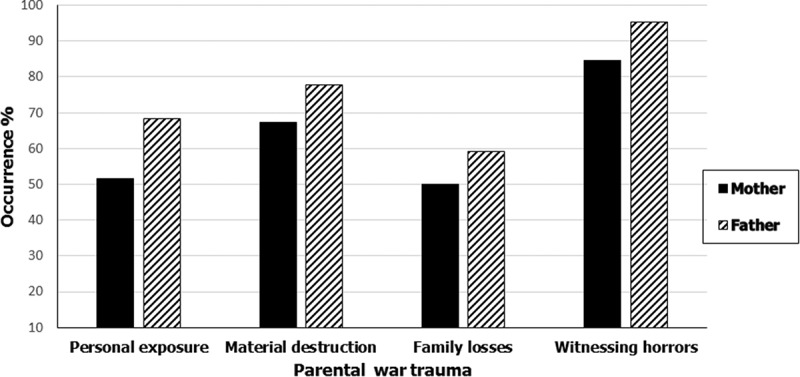
Occurrence (%) of war trauma among mothers and fathers.

Figure 1 shows the occurrence of the parents’ reported war trauma. Witnessing horrors was the most common war trauma among both fathers (96.2%) and mothers (89.2%). Furthermore, about half of the mothers reported personal exposure to trauma or family losses, and about 68.4% and 59.7% of fathers reported the same, respectively.

Pearson product–moment correlations among parental and child attachment, siblingship, and parenting are presented in Table 2. Parents’ secure attachment availability did not correlate with their children’s attachment styles. Further, children’s secure and preoccupied styles correlated significantly and positively, indicating that, in our sample, only avoidant attachment could be considered a genuinely insecure style. Children’s secure attachment correlated positively with warmth and intimacy and negatively with conflict and rivalry in sibling relations, and avoidant attachment was negatively correlated with warm and intimate siblingship and positively correlated with sibling conflict. Preoccupied attachment correlated positively with warm sibling relations. Both parents’ and children’s secure attachment were negatively correlated with emotional abuse and emotional neglect, and children’s avoidant attachment correlated negatively with these parenting practices. Intimacy among siblings was correlated negatively with parent- and child-reported emotional neglect, and warmth among siblings correlated negatively with parent-reported harsh parenting. Conflict and rivalry correlated positively with parent-reported harsh parenting, and rivalry in siblingship correlated positively with child-reported emotional neglect.

**Table 2.** Pearson’s product model correlations between family variables of the cluster analysis.

		1	2	3	4	5	6	7	8	9	10	11	12	13	14
Family attachment															
1	Maternal secure attachment														
2	Paternal secure attachment	0.52**													
3	Child secure attachment	0.09	0.08												
4	Child insecure: avoidant	−0.09	0.10	−0.38**											
5	Child insecure: ambivalent	0.04	0.08	0.28**	−0.48**										
Sibling relations															
6	Warmth	0.11	0.10	0.12*	−0.18**	0.14**									
7	Intimacy	0.09	0.10	0.14*	−0.13*	0.09	0.38**								
8	Conflict	−0.17**	−0.17*	−0.22**	0.11*	−0.03	−0.14**	−0.11*							
9	Rivalry	−0.13	−0.13	−0.17**	0.03	−0.07	0.05	0.09	0.39**						
Negative parenting															
10	Emotional abuse (parents)	−0.38**	−0.38**	−0.20**	0.16**	−0.13**	−0.06	−0.06	0.13*	0.13*					
11	Emotional neglect (parents)	−0.34**	−0.32**	−0.20**	0.15**	−0.11*	−0.08	−0.13*	0.16**	0.15*	0.89***				
12	Harsh parenting (parents)	−0.13*	−0.07	−0.09	0.00	−0.04	−0.16*	−0.03	−0.01	−0.09	0.24**	0.26**			
13	Emotional abuse (child)	−0.13*	−0.19	−0.20**	0.18**	−0.12*	−0.07	−0.06	0.08	0.11	0.73***	0.66***	0.11		
14	Emotional neglect (child)	−0.16**	−0.19**	−0.19**	0.15**	−0.10	−0.09	−0.14*	0.09	0.12*	0.64***	0.73***	0.18**	0.83***	
15	Harsh parenting (child)	−0.09	−0.06	−0.07	0.10*	−0.06	−0.03	−0.07	0.02	0.04	0.10*	0.12*	0.08	0.16**	0.16**

*p < 0.05, **p < 0.01, ***p < 0.001 (two-tailed; *N* = 304–334).

### 3.2. Identified family types

According to the hierarchical cluster dendrogram, a four-cluster solution was selected because it covered all the data, showed sufficient entropy, and proposed clusters with relatively high numbers of members. The *K*-cluster analysis confirmed the four-class solution, and Table 3 presents the means, standard errors, and analysis of variance (ANOVA) statistics for the differences among the identified family types with respect to parental attachment, child attachment, siblingship, and negative parenting. The MANOVA test showed a highly significant fit [*F*_Wilks’ lamb__d__a_(45,785.057) = 24.05, *p* < 0.0001, *η* ^2^ = 0.58].

**Table 3.** Means (*M*) and standard errors (*SE*) of parental and child factors according to the family-type cluster membership.

	Security and positive relationships		Insecurity and negative relationships		Discrepant experiences		Moderate security and neutral relationships			
	*M*	*SE*	*M*	*SE*	*M*	*SE*	*M*	*SE*	*F* (3,279)	*Partial η*^2^
Family attachment										
Maternal secure attachment	4.84^a^	0.06	3.92^b^	0.09	4.75^a^	0.07	4.07^b^	0.06	40.37****	0.30
Paternal secure attachment	4.87^a^	0.06	3.91^b^	0.09	4.64^a^	0.07	4.05^b^	0.06	42.34****	0.31
Child attachment										
Secure attachment	3.64^a^	0.08	2.66^b^	0.09	2.80^b^	0.09	3.84^a^	0.08	20.95****	0.18
Insecure: avoidant	1.76^a^	0.09	2.20^b^	0.08	1.92^c^	0.09	1.76^a^	0.09	22.99****	0.20
Insecure: preoccupied	2.99^ac^	0.06	2.63^b^	0.06	2.90^c^	0.08	3.07^a^	0.09	14.69****	0.14
Siblingship										
Warmth	2.70^a^	0.07	2.19^b^	0.10	2.52^a^	0.08	2.70^a^	0.07	6.79****	0.07
Intimacy	2.31^a^	0.06	2.03^a^	0.10	2.16^a^	0.08	2.05^a^	0.06	3.11***	0.03
Conflict	1.17^a^	0.07	1.72^b^	0.10	1.32^a^	0.09	1.23^a^	0.07	6.69****	0.07
Rivalry	1.02^a^	0.08	1.62^b^	0.11	1.20^a^	0.09	1.01^a^	0.08	7.52****	0.07
Negative parenting										
Emotional abuse (parents)	1.47^a^	0.05	2.91^b^	0.08	1.60^a^	0.06	2.45^c^	0.05	123.85****	0.57
Emotional neglect (parents)	1.69^a^	0.06	2.99^b^	0.06	1.78^a^	0.06	2.54^c^	0.05	83.30****	0.47
Harsh parenting (parents)	2.98^a^	0.06	3.46^b^	0.09	3.07^ac^	0.07	3.30^bc^	0.07	8.69***	0.09
Emotional abuse (child)	1.22^a^	0.07	2.47^b^	0.11	3.15^c^	0.09	2.08^d^	0.08	101.30****	0.52
Emotional neglect (child)	1.28^a^	0.08	2.63^b^	0.12	3.16^c^	0.10	2.23^d^	0.09	85.34****	0.48
Harsh parenting (child)	2.94^a^	0.07	3.64^b^	0.11	3.56^b^	0.09	3.01^a^	0.09	15.62****	0.14

Distribution of family types: security and positive relationships, *n* = 102; insecurity and negative relationships, *n* = 44; discrepant experiences, *n* = 65; moderate security and neutral relationships, *n* = 71.

^a,b,c,d^Different subscripts within columns indicate statistically significant differences between the family types, p <0.05.

*p <0.05, **p <0.01, ***p <0.001, ****p < 0.0001.

The four distinct family types showed unique attachment, sibling relationships, and negative parenting practices. About a third belonged to the first family type class, ‘security and positive relationships’ (*n* = 102; 36%), characterized by high parental and child attachment security and low avoidant attachment among children. Sibling relations showed high levels of warmth and intimacy and low levels of conflict and rivalry. Further, parent- and child-perceived harsh parenting were the lowest in this family type. The second family type class was labelled ‘insecurity and negative relationships’ (*n* = 44; 15.6%) and was characterized by low parental and child attachment security and high avoidant attachment among children. However, the children’s preoccupied attachment was lowest in these families. Sibling conflicts and rivalry were most common in this family type, and warmth was very low. Both parent- and child-reported harsh parenting was very high. The third family type class was called ‘discrepant experiences’ (*n* = 65; 23.0%) and was characterized by both negative and positive family relationships, although these were perceived differently by parents and children. Most notably, parents reported very low levels of harsh parenting, whereas children perceived high levels of harsh parenting. Children’s attachment patterns showed very high avoidance, low levels of security, and relatively high levels of preoccupied attachment, whereas parents showed high levels of secure attachment. Finally, about a quarter belonged to the fourth family type class, ‘moderate security and neutral relationships’ (*n* = 71; 25.2%). Like the parents in the insecurity and negative relationships family type, parents in this class showed low levels of secure attachment. By contrast, children showed high secure and preoccupied attachment and low avoidant attachment, similarly to children in the security and positive relationships families. Furthermore, the quality of siblingship seemed to be comparable to that in the security and positive relationships families. Both parent- and child-reported harsh parenting was moderate, between the levels reported by the security and positive relationships families and the insecurity and negative relationships families.

### 3.3. War trauma and family types

The MANOVA results revealed that family types differed significantly in terms of the severity of parental war trauma [*F*_Wilks’ lambda_(24,685.073) = 2.39, *p* < 0.0001, *η* ^2^ = 0.08]. The significant ANOVAs and Tukey-b post-hoc tests showed that fathers in insecurity and negative relationships families reported more material destruction [*F*(3,243) = 5.04, *p* < 0.002, *η* ^2^ = 0.06] than fathers in other types of families, and more personal exposure to trauma [*F*(3,243) = 5.50, *p* < 0.001, *η* ^2^ = 0.06] than fathers in security and positive relationship and discrepant experience families. Fathers in insecurity and negative relationships families also reported high levels of family losses (*F*(3,243) = 3.01, *p* < 0.03, *η* ^2^ = 0.04], but the post-hoc test was not significant. Mothers in the insecurity and negative relationships families also reported more personal exposure to trauma [*F*(3,243) = 9.59, *p* < 0.0001, *η* ^2^ = 0.11] than mothers in all other families, and more material destruction [*F*(3,243) = 2.65, *p* < 0.050, *η* ^2^ = 0.03] than mothers in the security and positive relationship and discrepant experience families.

### 3.4. Family types and children’s mental health

The MANCOVA results showed significant associations between family type and children’s mental health [*F*_Wilks’ lambda_(18,766 989) = 4.90, *p* < 0.0001, *η* ^2^ = 0.10]. Child gender was a significant covariant [*F*_Wilks’ lambda_(6,271.00) = 2.71, *p* < 0.01, *η* ^2^ = 0.06], but children’s own war trauma did not differ significantly across family types. We added the child gender and family type interaction terms in the analysis to check whether associations between family type and mental health were gender specific, but the interaction effects were non-significant.

Table 4 shows the means, standard errors, analysis of covaiance (ANCOVA) statistics, and post-hoc tests. The results revealed that children in the security and positive relationships families showed lower levels of externalizing symptoms (reported by both parents and children) and lower levels of parent-reported internalizing and child-reported depressive symptoms than children in other family types. Children’s self-reported internalizing symptoms did not, however, differ significantly from families with discrepant experience. Children in insecurity and negative relationships families showed higher levels of internalizing, externalizing, and depressive symptoms than children in security and optimal relationships families. However, the levels of mental health problems among children in the insecurity and negative relationships families did not differ significantly from those of children in families with discrepant experience and moderate security and neutral relationships (indicated by parent-reported externalizing and child-reported internalizing and depressive symptoms), families with discrepant experience (indicated by child-reported externalizing symptoms), and families with moderate security and neutral relationships (indicated by parent-reported internalizing symptoms). Children’s PTSD symptoms did not differ among the family types.

**Table 4.** Means (*M*) and standard errors (*SE*) of children’s mental health and post-traumatic cognitions according to family type.

	Security and positive relationships		Insecurity and negative relationships		Discrepant experiences		Moderate security and neutral relationships			
	*M*	*SE*	*M*	*SE*	*M*	*SE*	*M*	*SE*	*F* (3,279)	*Partial η^2^*
Children’s mental health										
Internalizing (parents)	6.12^a^	0.26	8.36^b^	0.40	6.15^a^	0.33	8.06^b^	0.31	13.85****	0.13
Externalizing (parents)	5.19^a^	0.29	8.04^b^	0.44	6.35^cb^	0.36	7.36^b^	0.34	13.27****	0.12
Internalizing (child)	6.63^a^	0.30	8.79^b^	0.46	7.78^ab^	0.38	7.97^b^	0.36	6.20****	0.06
Externalizing (child)	4.12^a^	0.32	7.09^b^	0.49	5.92^bc^	0.40	5.49^c^	0.39	9.76****	0.09
Depressive symptoms	10.59^a^	0.42	14.28^b^	0.64	12.60^b^	0.52	13.34^b^	0.52	10.05****	0.10
PTSD symptoms	28.86	1.00	30.43	1.46	29.20	1.28	28.46	1.19	0.64	0.01
Children’s post-traumatic cognitions										
Feeble person in scary world	23.93^a^	0.55	27.36^b^	0.85	27.46^b^	0.69	26.51^b^	0.66	7.12****	0.07
Permanent negative change	26.75^a^	0.68	30.71^b^	0.99	29.28^ab^	0.85	29.38^ab^	0.67	4.31**	0.04

Distribution of family types: security and positive relationships, *n* = 102; insecurity and negative relationships, *n* = 44; discrepant experiences, *n* = 65; moderate security and neutral relationships, *n* = 71.

PTSD, post-traumatic stress disorder.

^a, b, c^Different subscripts within columns indicate statistically significant differences between the family types, p <0.05.

*p <0.05, **p <0.01, ***p <0.001, ****p < 0.0001.

### 3.5. Family types and children’s post-traumatic cognitions

The MANCOVA results showed significant associations between family type and children’s PTCs [*F*_Wilks’ lambda_(6,550.00) = 3.71, *p* < 0.001, *η* ^2^ = 0.04]. Children’s gender and own war trauma were significant covariates [*F*_Wilks’ lambda_(6,275.00) = 5.49, *p* < 0.005, *η* ^2^ = 0.04 and *F*_Wilks’ lambda_(6,275.00) = 8.95, *p* < 0.0001, *η* ^2^ = 0.06, respectively]. Yet, the added interaction terms between child gender and family types, or between war trauma and family types showed non-significant results, indicating that family type associations with PTCs were not gender specific and did not depend on the severity of the children’s war trauma.

Means, standard errors, ANCOVA statistics, and post-hoc tests of the appraisals of being a feeble person in a scary world and permanent negative change are shown in Table 4. The results revealed that children in security and positive relationships families showed significantly lower levels of feeble person in a scary world appraisals than children in all other family types, as well as a lower level of permanent negative change appraisals than children in insecurity and negative relationships families. However, the levels of permanent negative change did not differ significantly either between the discrepant experiences and moderate security and neutral relationships families or between these families and security and insecurity families.

## 4. Discussion

The aim of the current study was to identify different family types among Palestinians living in the politically unstable and militarily dangerous Gaza Strip. Similarly to research on family systems in peaceful societies (Johnson, 2010; Lindblom et al., 2014), the study revealed multiple family dynamics. Among Palestinians, a secure family type with warm siblingship and optimal parenting practices was more than twice as common as an insecure family type with very negative relational patterns (36% vs 16%). The discrepant experiences and moderate security and neutral relationships family types, both incorporating different perceptions of reality between parents and children, accounted for a quarter of Palestinian families. Family type was found to be decisive for both children’s mental health (indicated by externalizing, internalizing, and depressive symptoms) and their ways of processing traumatic experiences (indicated by PTCs). As expected, secure and positive relationships families provided the best resources for both good mental health and effective processing of traumatic experiences. Yet, not only families with insecure and negative relationships, but also families with discrepant experiences were harmful for children’s well-being.

### 4.1. Family types in war conditions

The finding of a high prevalence of families with security and optimal sibling and parent–child relations is important when considering the life-threatening living conditions of the participating Palestinian families. Both the children and parents in these families reported high attachment security, and the children also exhibited low avoidance. The results concur with research empirically confirming the emergence of the classic family systems (Minuchin, 1974) and found that cohesive, balanced, and secure family types tend to form the majority (Lindblom et al., 2014; Johnson, 2010). The phenomenon of highly secure families in conditions of severe unsafety may indicate parents’ commitment and motivation to protect their children’s development and well-being in war zones. A similar resilience-enhancing rationale has been found among families suffering transgenerational trauma (Fossion et al., 2015).

In discrepant experience families, children and parents experienced different realities; or, at least, they perceived their attachment and parent–child relations in nearly opposite ways. Children showed very high avoidance, whereas parents perceived a high level of secure attachment relationships. Further, children experienced severe parental emotional abuse and emotional neglect, whereas parents reported very low levels of these negative parenting practices. These discrepancies between generations may reflect specific contextual political and military histories in the participating families. Palestinian children and youth are active in the national struggle for independence, often facing life-endangering military confrontations and sharing adult responsibilities (Qouta et al., 2008). In family therapeutic terms, children’s struggle for national safety can be described as a role reversal (Minuchin, 1974), which might be reflected in the discrepant family dynamics. Long-lasting military conflicts can contribute to discrepant experiences between parents and children.

In the moderate security and neutral relationships families, parents showed low levels of secure attachment responses, whereas children showed high levels of secure attachment responses. Attachment styles are suggested to be ‘inheritable’ because most secure mothers also have secure infants, toddlers, and, to some extent, adolescents (Van Ijzendoorn & Bakermans-Kranenburg, 1996). Thus, the discrepant experience and moderate security and neutral relationships families were anomalies, as mothers and fathers and children differed in their attachment security. The attachment patterns in the moderate security and neutral relationships families also contradicted attachment theory in that children showed high levels of both secure and preoccupied attachment responses. Again, this anomaly may be rooted in war and threats to life. It is possible that clinging to parents, friends, and siblings may be a survival skill for children in dangerous environments. Children’s preoccupied attachment, characterized by excessive dependency on parents or other people and high anxiety regarding their own security, can be understood as a functional or matching response to arbitrary, unpredictable, and emotionally oscillating parenting (Bowlby, 1988). We may speculate that the preoccupied style is a matching attachment in war conditions. The finding that children in insecurity and negative relationships families showed the lowest level of the preoccupied attachment supports this suggestion.

The patterns of the insecurity and negative relationships families echo those described in earlier literature on war- and trauma-affected families. Both parents and children reported low secure attachment orientations, and children also reported avoidant attachment. Siblingships incorporated conflicts, rivalry, and very little warmth, and parents and children both reported high levels of emotional abuse, neglect, and harsh parenting. Earlier research on war-affected families, especially veteran families, has reported that parental trauma has negative impacts on intimate marriage relations and the mental health of both parents and their children (Dekel & Monson, 2010). Similarly, in this study of civilian families, both material destruction and parents’ exposure to war trauma involving torture and ill-treatment were more common in insecure and negative relationships families than in other family types.

### 4.2. Family type and child well-being

The quality of family attachment and other relationships contributed significantly to children’s mental health, which is in line with ample earlier evidence (Sturge-Apple, Davies, Cicchetti, & Fittoria, 2014). Our findings showed that children experienced symptoms of heightened aggression, anxiety, and depression in families characterized by insecure attachment, sibling conflicts, and negative parenting. In contrast, in families characterized by parental secure attachment availability, warm siblingship, and optimal parenting, children showed lower levels of such symptoms. Similar to research in peaceful countries (Lindblom et al., 2017), our results showed that discrepant family dynamics were detrimental for children’s mental health, as children in the discrepant experiences families were just as likely to suffer from externalizing (e.g. aggression) and internalizing (e.g. depression) symptoms as children in the insecurity and negative relationships families, which were considered the most unfortunate families. The finding concurs with evidence that parental child-rearing practices that communicate opposite messages and family atmospheres that involve disagreements can endanger children’s healthy development and well-being (Crittenden & Dallos, 2009). Perhaps unexpectedly, PTSD symptoms did not vary according to family type. However, this result concurs with the understanding that the severity, timing, and type of traumatic experiences are the most important determinants of the severity of PTSD (Lambert, Holzer, & Hasbun, 2014).

Families provide different resources to their children to support them in optimal cognitive–emotional processing of trauma. Our results suggest that the quality of family attachment and sibling and parenting relations may be decisive in how children appraise and cognitively process their traumatic experiences, here indicated by PTCs. Children in three identified family types – insecurity and negative relationships families, discrepant experiences families, and moderately secure and neutral relationships families – were prone to using dysfunctional cognitive appraisals. These children felt that they were feeble and fragile persons in a frightening and dangerous world and that trauma had negatively marked them forever. This finding is alarming, as functional regulating, appraising, and reconstructing traumatic experiences are often preconditions for recovery from war trauma. Only the secure families with optimal relations were able to promote their children’s effective, functional, and robust processing of traumatic events, as indicated by their positive PTCs.

The findings concerning PTCs are important, as they support the idea that interventions for war-affected children should also enhance secure family relationships, including warm and supportive siblingships and parenting, to improve children’s emerging abilities to process, deal, and cope with war trauma. It is also necessary to learn about the rich variety of attachment-informed family interventions (e.g. Lieberman, 2003; Toth & Cicchetti, 2011) when helping families in war conditions. Cognitive-behavioural therapies (CBTs) and interventions are commonly recommended for war-affected children (Betancourt, Meyers-Ohki, Charrow, & Tol, 2013). The core healing elements in these treatments involve improving appraisals, attention, memories, and world views. Therefore, when tailoring CBTs, it is necessary to consider the importance of family influences on children’s PTCs. We recommend that the intervention studies should include attachment style or family type as a moderator in their effectiveness analyses. Many current school- or group-based interventions for war-affected children rely, either explicitly or implicitly, on the children’s ability and willingness to trust others and expect them to change their dysfunctional cognitions in a trustful therapeutic atmosphere. However, not all children are capable of that kind of beneficial interaction. Therefore, information on family attachment styles and other relational qualities could support a better understanding of the individual differences in the needs and timing of building trust with a therapist or a peer group. Such an understanding is crucial for tailoring cognitive intervention work towards better mental health.

### 4.3. Limitations of the study

Our study has a few methodological and theoretical limitations. First, we used self-reported attachment measurements. It would have been preferable to employ, for instance, the Adult Attachment Interview, with its dynamic dimensions of coherence, idealization, and unresolved traumatic past. The children’s depressive and PTSD symptoms were also self-reported. Although we used multi-source reports to measure children’s externalizing and internalizing symptoms, clinical interviews would be more accurate and insightful. Secondly, statistically, approaches more sophisticated than cluster analysis would support a more robust identification of family types. For instance, latent mixture modelling or latent profile analysis could provide more statistical criteria for determining the number of naturally occurring subgroups or latent classes using structural equation modelling. Thirdly, parenting quality was conceptualized very negatively in this research; that is, as harsh parenting and emotional abuse and neglect. The choice was based on the literature on transgenerational trauma, which can be transmitted by malevolent relations or harsh parenting (Yehuda & Bierer, 2008). Yet, a measure that covers both the positive and the negative dimensions of parenting styles would be more legitimate, as traumatic stress and threats to life activate both kinds of parental responses. Fourthly, the reliabilities of some variables were low (less than 0.70), and the attachment style dimensions were particularly inconsistent. This warrants caution in interpreting the results. Fifthly, our study setting was cross-sectional; however, to fully understand the impacts of war on family dynamics and their combined effects on child well-being, a longitudinal setting is required. Finally, we used the children’s reports of war trauma only as covariates in our analyses, whereas critics may suggest that these are a natural part of family war trauma.

Despite these shortcomings, the study provides a comprehensive view of family life in conditions of war and military violence. It contributes to the family-related trauma research by showing the importance of a whole family approach that includes parents, children, siblings, and societal context (Riggs & Riggs, 2011). It would be fruitful to further analyse the perceptions, experiences, and mental health of mothers, fathers, and multiple siblings living in war areas in order to further develop the body of family systems-informed trauma research. From a human rights perspective, children should feel safe and secure in their homes, schools, and communities. Yet, in the life-endangering conditions of war, parents are often burdened by an overwhelming sense of responsibility for their children’s security (Cummings et al., 2016; Punamäki, 2014). When the larger society does not fulfil its protective role, the quality of family relations becomes highly important. Identifying family types according to the quality of parental attachment availability and children’s attachment security, siblingship, and parenting qualities may be a fruitful approach to developing effective interventions to help families to endure during war. The results reveal differences in families’ capabilities to provide resources to help their children maintain their mental health and functionally process traumatic experiences.

## References

[CIT0001] Attanayake V., McKay R., Joffres M., Singh S., Burkle F. Jr., & Mills E. (2009). Prevalence of mental disorders among children exposed to war: A systematic review of 7,920 children. , 25(1), 4–15. 10.1080/13623690802568913 19413154

[CIT0002] Barajas-Gonzalez R. G., & Brooks-Gunn J. (2014). Income, neighborhood stressors, and harsh parenting: Test of moderation by ethnicity, age, and gender. , 28(6), 855–866. 10.1037/a0038242 25383794

[CIT0003] Bergman L. R, & Magnusson D. (1997). A person-oriented approach in research on developmental psychopathology. , 9(2), 291–319. 10.1017/s095457949700206x 9201446

[CIT0004] Besser A., & Neria Y. (2010). The effects of insecure attachment orientations and perceived social support on posttraumatic stress and depressive symptoms among civilians exposed to the 2009 Israel–Gaza war: A follow-up Cross-Lagged panel design study. , 44(3), 335–341.

[CIT0005] Betancourt T. S., Meyers-Ohki S., Stulac S. N., Barrera A. E., Mushashi C., & Beardslee W. R. (2011). Nothing can defeat combined hands (Abashize hamwe ntakibananira): Protective processes and resilience in Rwandan children and families affected by HIV/AIDS. , 73(5), 693–701. 10.1016/j.socscimed.2011.06.053 PMC3162991 21840634

[CIT0006] Betancourt T. S., Meyers-Ohki S. E., Charrow A. P., & Tol W. A. (2013). Interventions for children affected by war: An ecological perspective on psychosocial support and mental health care. , 21(2), 70–91. 10.1097/HRP.0b013e318283bf8f PMC4098699 23656831

[CIT0007] Birleson P., Hudson I., Grey-Buchanan D., & Wolff S. (1987). Clinical evaluation of a self-rating scale for depressive disorder in childhood (depression self-rating scale). , 28, 43–60. 10.1111/j.1469-7610.1987.tb00651.x 3558538

[CIT0008] Blass R. B., & Blatt S. J. (1992). Attachment and separateness. A theoretical context for the integration of object relations theory with self psychology. , 47, 189–203. 1289929

[CIT0009] Bowlby J. (1969/1982). (Vol. 1). New York: Basic Books.

[CIT0010] Bowlby J. (1988). . New York: Basic Books.

[CIT0011] Bryant R. A. (2016). Social attachments and traumatic stress. , 7, 29065. 10.3402/ejpt.v7.29065 PMC4800287 26996531

[CIT0012] Cohen E., Zerach G., & Solomon Z. (2011). The implication of combat-induced stress reaction, PTSD, and attachment in parenting among war veterans. , 25(5), 688–698. 10.1037/a0024065 21639634

[CIT0013] Coyne J. C., Downey G., & Boergers J. (1992). Depression in families: A systems perspective In Cicchetti D. (Ed.), (pp. 211–249). Rochester, NY: University of Rochester.

[CIT0014] Crittenden P. M., & Dallos R. (2009). All in the family: Integrating attachment and family systems theories. , 14(3), 389–409. 10.1177/1359104509104048 19515755

[CIT0015] Cummings E. M., Merrilees C. E., Schermerhorn A. C., Goeke-Morey M. C., Shirlow P., & Cairns E. (2011). Longitudinal pathways between political violence and child adjustment: The role of emotional security about the community in Northern Ireland. , 39(2), 213–224. 10.1007/s10802-010-9457-3 PMC3677545 20838875

[CIT0016] Cummings E. M., Taylor L. K., Merrilees C. E., Goeke-Morey M. C., & Shirlow P. (2016). Emotional insecurity in the family and community and youth delinquency in Northern Ireland: A person-oriented analysis across five waves. , *57*(1), 47–54. 10.1111/jcpp.12427 PMC4644723 25981614

[CIT0017] Daud A., Skoglund E., & Rydelius P.-A. (2005). Children in families of torture victims: Transgenerational transmission of parents’ traumatic experiences to their children. , 14(1), 23–32.

[CIT0018] Davies P. T., & Cicchetti D. (2004). Toward an integration of family systems and developmental psychopathology approaches. , 16(3), 477–481. 10.1017/s0954579404004626 15605621

[CIT0019] De Haene L, Grietens H, & Verschueren K (2010). Adult attachment in the context of refugee traumatisation: the impact of organized violence and forced separation on parental states of mind regarding attachment. , 12(3), 249-264. 10.1080/14616731003759732 20473796

[CIT0020] Dekel R., & Monson C. M. (2010). Military-related post-traumatic stress disorder and family relations: Current knowledge and future directions. , 15(4), 303–309.

[CIT0021] Dubow E. F., Boxer P., Huesmann L. R., Shikaki K., Landau S., Gvirsman S. D., & Ginges J. (2009). Exposure to conflict and violence across contexts: Relations to adjustment among Palestinian children. , 39(1), 103–116. 10.1080/15374410903401153 PMC2856124 20390802

[CIT0022] Dunn J., Slomkowski C., & Beardsall L. (1994). Sibling relationships from the preschool period through middle childhood and early adolescence. , 30, 315–324. 10.1111/j.1469-7610.1994.tb01736.x 8195308

[CIT0023] Dyregrov A., Gjestad R., & Raundalen M. (2002). Children exposed to warfare: A longitudinal study. , 15(1), 59–68. 10.1023/A:1014335312219 11936723

[CIT0024] Ehlers A., & Clark D. M. (2000). A cognitive model of posttraumatic stress disorder. , 38, 319–345. 10.1016/s0005-7967(99)00123-0 10761279

[CIT0025] Ehlers A., Mayou R. A., & Bryant B. (2003). Cognitive predictors of posttraumatic stress disorder in children: Results of a prospective longitudinal study. , 41, 1–10. 10.1016/s0005-7967(01)00126-7 12488116

[CIT0026] Ein-Dor T., Doron G., Solomon Z., Mikulincer M., & Shaver P. R. (2010). Together in pain: Attachment-related dyadic processes and posttraumatic stress disorder. , 57(3), 317–327. 10.1037/a0019500 21133582

[CIT0027] Feldman R., Vengrober A., Eidelman-Rothman M., & Zagoory-Sharon O. (2013). Stress reactivity in war-exposed young children with and without posttraumatic stress disorder: Relations to maternal stress hormones, parenting, and child emotionality and regulation. , 25(4pt1), 943–955. 10.1017/S0954579413000291 24229541

[CIT0028] Finnegan R. A, Hodges E. V. E., & Perry D. G (1996). Preoccupied and avoidant coping during middle childhood. , 67(4), 1318-1328.

[CIT0029] Flykt M., Kanninen K., Sinkkonen J., & Punamäki R.-L. (2010). Maternal depression and dyadic interaction: The role of maternal attachment style. , 19, 530–550.

[CIT0030] Foa E. B., Ehlers A., Clark D. M., Tolin D. F., & Orsillo S. M. (1999). The posttraumatic cognitions inventory (PTCI): Development and validation. , 11(3), 303–314.

[CIT0031] Fossion P., Leys C., Vandeleur C., Kempenaers C., Braun S., Verbanck P., & Linkowski P. (2015). Transgenerational transmission of trauma in families of Holocaust survivors: The consequences of extreme family functioning on resilience, sense of coherence, anxiety and depression. , 171, 48–53. 10.1016/j.jad.2014.08.054 25285898

[CIT0032] Freedman S. A., Gilad M., Ankri Y., Roziner I., & Shalev A. Y. (2015). Social relationship satisfaction and PTSD: Which is the chicken and which is the egg? , 6, 28864. 10.3402/ejpt.v6.28864 PMC4696463 26684986

[CIT0033] Frewen P., Brown M., DePierro J., D’Andrea W., & Schore A. (2015). Assessing the family dynamics of childhood maltreatment history with the Childhood Attachment and Relational Trauma Screen (CARTS). , 6, 27792. 10.3402/ejpt.v6.27792 PMC4524890 26243548

[CIT0034] Goodman R (1997). The strengths and difficulties questionnaire: A research note. , 38(5), 581–586. 10.1111/j.1469-7610.1997.tb01545.x 9255702

[CIT0035] Huemer J., Nelson K., Karnik N., Völkl-Kernstock S., Seidel S., Ebner N., … Skala K. (2016). Emotional expressiveness and avoidance in narratives of unaccompanied refugee minors. , 7, 29163. 10.3402/ejpt.v7.29163 PMC4783431 26955827

[CIT0036] Johnson V. K. (2010). From early childhood to adolescence: Linking family functioning and school behavior. , 59(3), 313–325. 10.1111/j.1741-3729.2010.00604.x PMC3023307 21258653

[CIT0037] Kerig P. K. (2005). Revisiting the construct of boundary dissolution. , 5, 5–42.

[CIT0038] Kerns K. A., Klepac L., & Cole A. K. (1996). Peer relationships and preadolescents’ perceptions of security in the child-mother relationship. , 32(3), 457–466.

[CIT0039] Kerns K. A., Tomich P. L., Aspelmeier J. E., & Contreras J. M. (2000). Attachment-based assessments of parent–Child relationships in middle childhood. , 36(5), 614–626. 10.1037/0012-1649.36.5.614 10976601

[CIT0040] Khamis V (2000). Child psychological maltreatment in palestinian families. , 24(8), 1047 –1059. 10.1016/s0145-2134(00)00157-5 10983815

[CIT0041] Lambert J. E., Holzer J., & Hasbun A. (2014). Association between parents’ PTSD severity and children’s psychological distress: A meta-analysis. , 27(1), 9–17. 10.1002/jts.21891 24464491

[CIT0042] Lieberman A. F (2003). The treatment of attachment disorder in infancy and early childhood: reflections from clinical intervention with later-adopted foster care children. , 5(3), 279 –282. 10.1080/14616730310001596133 12944223

[CIT0043] Lindblom J., Flykt M., Tolvanen A., Vänskä M., Tiitinen A., Tulppala M., & Punamäki R.-L. (2014). Dynamic family system trajectories from pregnancy to child’s first year. , 76(4), 796–807.

[CIT0044] Lindblom J., Vänskä M., Flykt M., Tolvanen A., Tiitinen A., Tulppala M., & Punamäki R.-L. (2017). From early family systems to internalizing symptoms: The role of emotion regulation and peer relations. , 31(3), 316–326. 10.1037/fam0000260 27854439

[CIT0045] Masten A. S., & Monn A. R. (2015). Child and family resilience: A call for integrated science, practice, and professional training. , 64(1), 5–21.

[CIT0046] Meiser-Stedman R. (2002). Towards a cognitive-behavioral model of PTSD in children and adolescents. , 5(4), 217–232. 10.1023/a:1020982122107 12495267

[CIT0047] Meiser‐Stedman R., Smith P., Bryant R., Salmon K., Yule W., Dalgleish T., & Nixon R. D. V. (2009). Development and validation of the Child Post‐Traumatic Cognitions Inventory (CPTCI). , 50, 432–440. 10.1111/j.1469-7610.2008.01995.x 19338628

[CIT0048] Minuchin S. (1974). . Cambridge, MA: Harvard University Press.

[CIT0049] Montgomery E. (2004). Tortured families: A coordinated management of meaning analysis. , 43(3), 349–371. 10.1111/j.1545-5300.2004.00027.x 15386959

[CIT0050] Montgomery E. (2011). Trauma, exile and mental health in young refugees. , Suppl(440), 1–46. 10.1111/j.1600-0447.2011.01740.x 21824118

[CIT0051] Montgomery E., & Foldspang A. (2005). Seeking asylum in Denmark: Refugee children’s mental health and exposure to violence. , 15(3), 233–237. 10.1093/eurpub/cki059 15923213

[CIT0052] Olson D. (2000). Circumplex model of marital and family systems. , 22, 144–167. 10.1111/j.1545-5300.1983.00069.x 6840263

[CIT0053] Pallini S., Baiocco R., Schneider B. H., Madigan S., & Atkinson L. (2014). Early child-parent attachment and peer relations: A meta-analysis of recent research. , 28(1), 118–123. 10.1037/a0035736 24512287

[CIT0054] Pemberton J. R., Kramer T. L., Borrego J. Jr., & Owen R. R. (2013). Kids at the VA? A call for evidence-based parenting interventions for returning veterans. , 10(2), 194–202. 10.1037/a0029995 23088402

[CIT0055] Punamäki R. L. (2014). Mental health and development among children living in violent conditions: Underlying mechanisms for promoting peace In Britto P., Leckman J. F., Panter–Brick C., & Salah R. (Eds.), (Vol. 15). J Lupp, series ed. Cambridge, MA: MIT Press.

[CIT0056] Punamäki R.-L., Qouta S., El Sarraj E., & Montgomery E. (2006). Psychological distress and resources among siblings and parents exposed to traumatic events. , 30(5), 385–397.

[CIT0057] Qouta S., Punamäki R.-L., Miller T., & El Sarraj E. (2008). Does war beget child aggression? Military violence, gender, age and aggressive behavior in two Palestinian samples. , 34, 231–244. 10.1002/ab.20236 17985361

[CIT0058] Qouta S. R., Palosaari E., Diab M., & Punamäki R.-L. (2012). Intervention effectiveness among war affected children: A cluster randomized controlled trial on improving mental health. , 25, 288–298. 10.1002/jts.21707 22648703

[CIT0059] Riggs S. A., & Riggs D. S. (2011). Risk and resilience in military families experiencing deployment: The role of the family attachment network. , 25(5), 675–687. 10.1037/a0025286 21875201

[CIT0060] Scheeringa M. S., & Zeanah C. H. (2001). A relational perspective on PTSD in early childhood. , 14(4), 799–815. 10.1023/A:1013002507972 11776426

[CIT0061] Schierholz A., Kruger A., Barenbrugge J., & Ehring T. (2016). What mediates the link between childhood maltreatment and depression? The role of emotion dysregulation, attachment, and attributional style. , 7, 32652. 10.3402/ejpt.v7.32652 PMC5084388 27790969

[CIT0062] Schnyder U., Ehlers A., Elbert T., Foa E. B., Gersons B. P., Resick P. A., … Cloitre M. (2015). Psychotherapies for PTSD: What do they have in common? , 6, 28186. 10.3402/ejpt.v6.28186 PMC4541077 26290178

[CIT0063] Sturge-Apple M. L., Davies P. T., Cicchetti D., & Fittoria M. G. (2014). A typology of interpartner conflict and maternal parenting practices in high-risk families: Examining spillover and compensatory models and implications for child adjustment. , 26(4 Pt 1), 983–998. 10.1017/S0954579414000509 24914564

[CIT0064] Tabachnick B., & Fidell L. (2007). (5th ed.). Boston, MA: Allyn & Bacon.

[CIT0065] Toth S. L., & Cicchetti D. (2011). Frontiers in translational research on trauma. , 23(2), 353–355. 10.1017/S0954579411000101 23786682

[CIT0066] Trickey D., Siddaway A. P., Meiser-Stedman R., Serpell L., & Field A. P. (2012). A meta-analysis of risk factors for post-traumatic stress disorder in children and adolescents. , 32(2), 122–138. 10.1016/j.cpr.2011.12.001 22245560

[CIT0067] Turunen T., Haravuori H., Punamaki R. L., Suomalainen L., & Marttunen M. (2014). The role of attachment in recovery after a school-shooting trauma. , 5, 22728. 10.3402/ejpt.v5.22728 PMC4082197 25018861

[CIT0068] UN (2009). Human rights in Palestine and other occupied Arab territories: Report of the United Nations fact-finding mission on the Gaza conflict. New York, NY: UN General Assembly Retrieved from http://www2.ohchrorg/english/bodies/hrcouncil/docs/12session/A-HRC-12-48.pdf

[CIT0069] UN-OCHA (2009). . East Jerusalem, UN: Office for the Coordination of Humanitarian Affairs occupied Palestinian territory.

[CIT0070] Van Ee E., Kleber R. J., Jongmans M. J., Mooren T. T., & Out D. (2016). Parental PTSD, adverse parenting and child attachment in a refugee sample. , 18(3), 273–291. 10.1080/14616734.2016.1148748 26982876

[CIT0071] Van IJzendoorn M, & Bakermans-Kranenburg M. J (1996). Attachment representations in mothers, fathers, adolescents, and clinical groups: a meta-analytic search for normative data. , 64(1), 8 –14. 10.1037//0022-006x.64.1.8 8907080

[CIT0072] Van Ijzendoorn M. H., Bakermans-Kranenburg M. J., & Sagi-Schwartz A. (2003). Are children of Holocaust survivors less well-adapted? A meta-analytic investigation of secondary traumatization. , 16(5), 459–469. 10.1023/A:1025706427300 14584630

[CIT0073] Walsh F (2007). Traumatic loss and major disasters: strengthening family and community resilience. , 46(2), 207 –227. 10.1111/j.1545-5300.2007.00205.x 17593886

[CIT0074] Yehuda R., & Bierer L. M. (2008). Transgenerational transmission of cortisol and PTSD risk. , 167, 121–135. 10.1016/S0079-6123(07)67009-5 18037011

[CIT0075] Zerach G., Greene T., Ein-Dor T., & Solomon Z. (2012). The relationship between posttraumatic stress disorder symptoms and paternal parenting of adult children among ex-prisoners of war: A longitudinal study. , 26(2), 274–284. 10.1037/a0027159 22309816

